# Physician-Scientists in Environmental Health

**DOI:** 10.1289/ehp.113-a796

**Published:** 2005-12

**Authors:** David A. Schwartz

**Affiliations:** Director, NIEHS and NTP, E-mail: david.schwartz@niehs.nih.gov

I believe that the physician-scientist represents an endangered species in biomedical research, and is absolutely essential to achieving our vision for the NIEHS. As a physician-scientist, my thoughts on this topic are somewhat biased and reflect my parochial views, as well as the more reasoned concerns raised by others (Goldstein and Brown 1997; Ley and Rosenberg 2005; Rosenberg 2000; Wyngaarden 1979; Zerhouni 2005), on the fragile future of our species. Despite this admonition, I believe that our field will benefit enormously by expanding our cadre of physicians committed to the science of environmental health. While many people with whom I’ve talked emphasize the “E” in NIEHS, I think we need to pay more attention to the “H” in both NIEHS and NIH.

In general, the motivation that drives the quest for new knowledge in biomedical research is decidedly different for the physician-scientist. Although I spend most of my time in the research arena, the reason that I’m involved in research and the type of research that I do is largely inspired by my patients with unexplainable illnesses. The very question that patients and families frequently ask me—why one person (when challenged with an environmental agent) develops an illness while another person remains healthy—represents the focus of my research and has led me to the NIEHS. In fact, the longer I practice medicine (25 years and counting), the more I realize how much we don’t know about the science of medicine. We have so much more to understand about disease development, pathogenesis, treatment, and prevention. For the general public, this medical science gap usually doesn’t hit home until someone in the family develops a disease, or the message gets across from one of the research advocacy agencies, like Research America. The physician-scientist can help to identify the major opportunities in biomedical research that are likely to have the biggest impact on human health.

Our vision at the NIEHS, to use environmental sciences to understand human disease and to improve human health, requires that physician-scientists actively engage in the process. As we shift the focus of research supported by the NIEHS to emphasize human disease, the need for physician-scientists becomes compelling. While there are numerous examples of PhD-trained scientists who have had major effects on human health, MD-trained scientists are simply more familiar with the varied manifestations of human disease. Moreover, physicians bring the bedside to the science through their experiences, such as the clear memory of the patient who responded in an unusual way, which highlights some of the research opportunities in medicine. In this context, physicians have the unique ability to focus their research on scientific questions that are clinically relevant. While physician-scientists may lead scientific projects or teams studying a particular disease, it is just as likely that they will serve as the “glue,” helping a group of basic or public health investigators to focus their interests on clinically relevant areas of human pathophysiology.

As we conceive the future for the NIEHS, we need to consider the role of the physician-scientist in our institute. When we benchmark our institute to others at the NIH and consider independently funded investigators, it is clear that we have a much lower percentage of physicians as principal investigators than most of the institutes. I would suggest that we need to increase the percentage of physician-scientists at the NIEHS to at least 30% if we’re serious about shifting our focus to human health and disease.

To accomplish this, we are in the process of developing a number of extramural and intramural programs that focus on training, career development, independent research support, and specialized centers in integrative (translational) research. For instance, we recently established the Outstanding New Environmental Scientist (ONES) Award, an RFA that will fund first-time R01 recipients who are using environmental science to understand a human disease. In addition, we have decided to establish a Clinical Research Unit (CRU) within the Division of Intramural Research at the NIEHS. The CRU is being developed by Perry Blackshear, director of the Office of Clinical Research, and will be located on our campus at the NIEHS to afford the physician-scientist every opportunity to collaborate with basic and public health scientists. This will also enable our intramural scientists to take an interdisciplinary approach to broad themes in environmental health that cross methodological disciplines, such as reproductive health and epigenetics, neurosciences, immune-mediated diseases, and metabolism. The goal of this effort is to integrate basic, clinical, and public health science to have the biggest impact on human health.

If we keep our eye firmly fixed on the goal of understanding human disease and improving human health, we will surely have an impact. I believe that the physician-scientist will prove to be critical to the success of these efforts.

## Figures and Tables

**Figure:**
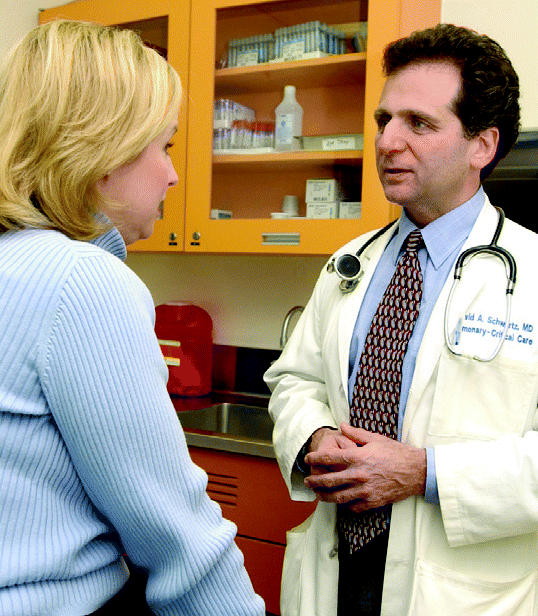


**Figure:**
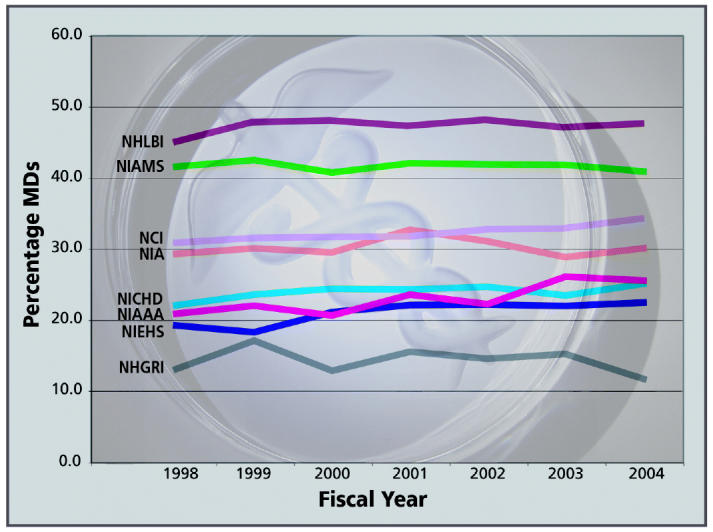
Principal Investigators with MDs at Benchmark NIH I/Cs
